# Tailoring of Magnetic Properties of NiO/Ni Composite Particles Fabricated by Pulsed Laser Irradiation

**DOI:** 10.3390/nano8100790

**Published:** 2018-10-05

**Authors:** Zaneta Swiatkowska-Warkocka, Alexander Pyatenko, Yoshiki Shimizu, Marcin Perzanowski, Arkadiusz Zarzycki, Benedykt R. Jany, Marta Marszalek

**Affiliations:** 1 Institute of Nuclear Physics Polish Academy of Sciences, PL-31342 Krakow, Poland; Marcin.Perzanowski@ifj.edu.pl (M.P.); Arkadiusz.Zarzycki@ifj.edu.pl (A.Z.); Marta.Marszalek@ifj.edu.pl (M.M.); 2 National Institute of Advanced Industrial Science and Technology, Tsukuba 305-8565, Japan; alexander.pyatenko@aist.go.jp (A.P.); shimizu.yoshiki@aist.go.jp (Y.S.); 3 Marian Smoluchowski Institute of Physics Jagiellonian University, 30-348 Krakow, Poland; benedykt.jany@uj.edu.pl

**Keywords:** nickel/nickel oxide composites, pulsed laser irradiation, exchange bias

## Abstract

We present NiO/Ni composite particles with face-centered cubic (fcc) structure prepared by a pulsed laser irradiation of NiO nanoparticles dispersed in liquid. The sizes of particles and the Ni content in NiO/Ni composites were controlled by tuning the laser parameters, such as laser fluence and irradiation time. We found that the weight fraction of Ni has a significant impact on magnetic properties of composite particles. Large exchange bias (H_EB_) and coercivity field (H_C_) were observed at 5 K due to the creation of heterojunctions at interfaces of ferromagnetic Ni and antiferromagnetic NiO. For the NiO/Ni composites with 80% of NiO we have observed the largest values of exchange bias (175 Oe) and coercive field (950 Oe), but the increase of Ni weight fraction resulted in the decrease of both H_C_ and H_EB_ values.

## 1. Introduction

Magnetic heterostructures composed of two or more components exhibit novel and unique properties induced by interactions between the constituents. Properties of multi-component particles depend not only on their size and structure, but can be also be tuned by their composition. Therefore, heterostructure particles have more extensive applications than single-component particles [1,2,3,4,5,6].

Ferromagnetic metal such as nickel (Ni) (Curie temperature Tc = 631 K) [7] is the object of growing interest arising from interesting physical properties and wide range of applications, e.g., in data storage, fuel cell electrodes and catalysis [8,9,10,11]. Nevertheless, the high over potential and large Tafel slope of Ni metal meanthat it is not an ideal water reductor or hydrogen evolution reaction catalyst [12,13]. Nickel oxide (NiO) is an attractive material, well known for its chemical stability and excellent optical and electrical properties [14,15,16,17]. Recently, NiO was explored as a new class of highly efficient electrocatalysts for oxygen evolution reaction (OER) [18], however, its poor electrical conductivity is the shortcoming. The bulk nickel oxide is antiferromagnetic with the Néel temperature of 523 K [19]. The NiO/Ni composites, as important ferromagnetic/antiferromagnetic material, are a good model for fundamental studies, and are promising composites for applications in catalysts, fuel-cell electrodes, magnetic memories [20,21,22,23,24]. 

It is well known that magnetic properties of composite particles are determined by the size, composition, microstructural features of both components, as well as the interface between them. Therefore, it is extremely important to control these features, especially at the stage of synthesis. Until now, only a few papers have reported in-depth studies on the effect of phase content of Ni in NiO/Ni composites on magnetic properties, especially exchange bias and coercivity field, of NiO/Ni composites particle [25,26,27,28]. We believe that the application of Ni/NiO composites will be wider after establishing the above relationship.

Nowadays, laser ablation, and laser irradiation in liquids area popular method for formation of metal and metal oxide particles because of their safety, simplicity and versatility [29,30,31,32,33,34,35,36,37].

In this study, we demonstrate a novel method for preparationand easy control of the size and composition of NiO/Ni composite particles by pulse laser irradiation of NiO nanoparticles dispersed in ethyl acetate. The raw (un-irradiated) oxide nanoparticles are reduced to a lower oxidized state, depending on laser fluence, or irradiation time and converted to nanocomposite submicron particles containing various phases with different oxidation states. Furthermore, we report influence of laser parameters on the size, phase composition, structure and microstructure of NiO/Ni composites. We show that by varying the laser parameters (laser fluence and/or irradiation time) we can control the phase ratio of Ni and NiO, and thus we can control the coercivity and exchange bias effect.

## 2. Materials and Methods

The NiO nanoparticles (<50 nm, nanopowder, Figure S1, Supporting Information) were purchased from Aldrich (Merck KGaA, Darmstadt, Germany). Raw nanoparticles of NiO (0.5 mM) dispersed in ethyl acetate (15 mL) were stirred and irradiated with the unfocused second harmonics (532 nm) of a yttrium aluminum garnet (Nd:YAG)laser operating at 10 Hz. Laser fluence was changed between 130 and 520 mJ/pulse·cm^2^, while irradiation time varied from 10 min to 90 min.

The crystal structure and composition of fabricated particles were determined by a powder X-ray diffraction (XRD; Panalytical X’Pert Pro, Malvern Panalytical Ltd., Royston, UK) with CuKα (Copper K-α) radiation. For XRD measurements the solution with synthesized particles was dried on a Si (100) substrate. XRD patterns were taken using the Cu Kα line at 1.54 Å. Cu radiation obtained at 40 kV and 30 mA was converted into a parallel beam by incident beam optics with 0.5° divergence slit, parabolic graded W/Si mirror with an equatorial divergence less than 0.05°, and 0.04 rad Soller slit collimator. The axial width of the incident beam was restricted by the incident beam mask to 5 mm. The diffracted beam path was equipped with an anti-scatter slit and 0.04 rad Soller slit collimator. The signal was collected by a solid state stripe detector with a graphite monochromator. The smallest step size of 2θ that could be defined with the optics was 0.0021° for a goniometer radius of 240 mm. NIST LaB6 (lanthanum hexaboride) line profile standard SRM660a was measured to determine the angular resolution of the instrument. Based on these measurements, the instrumental peak broadening of 0.05° was obtained for the 2θ range from 30° to 55° and all diffraction patterns were collected with a step size of 0.05°. Fullprof was used for peak fitting and to evaluate the lattice constants of the material. [38] Panalytical’s HighScore Plus software was used for quantitative phase analysis.

The morphology of particles was observed with a field emission scanning electron microscope (SEM microscope; FEI Quanta 3D FEG equipped with the EDAXEDS (Theromo Fisher Scientific, Hillsboro, OR, USA) detector system) and a transmission electron microscope (TEM; FEI Tecnai Osiris (Theromo Fisher Scientific, Hillsboro, OR, USA)). SEM micrographs were collected in two imaging modes: Secondary Electrons SE mode (topography contrast) and Backscattered Electrons BSE mode (atomic number contrast). High angle annular dark field scanning transmission electron microscopy (HAADF-STEM) images (atomic number contrast), and energy-dispersive X-ray (EDX) mapping were obtained using FEI Tecnai Osiris microscope at an accelerating voltage of 200 kV. The average particle size was determined by measuring the diameters of 200 particles randomly chosen from SEM images. A superconducting quantum interference device (SQUID; Quantum Design, MPMS XL, Quantum Design, Inc., San Diego, CA, USA) magnetometer was employed to measure the magnetic properties of nanocomposite particles.

## 3. Results and Discussion

**Figure 1:**
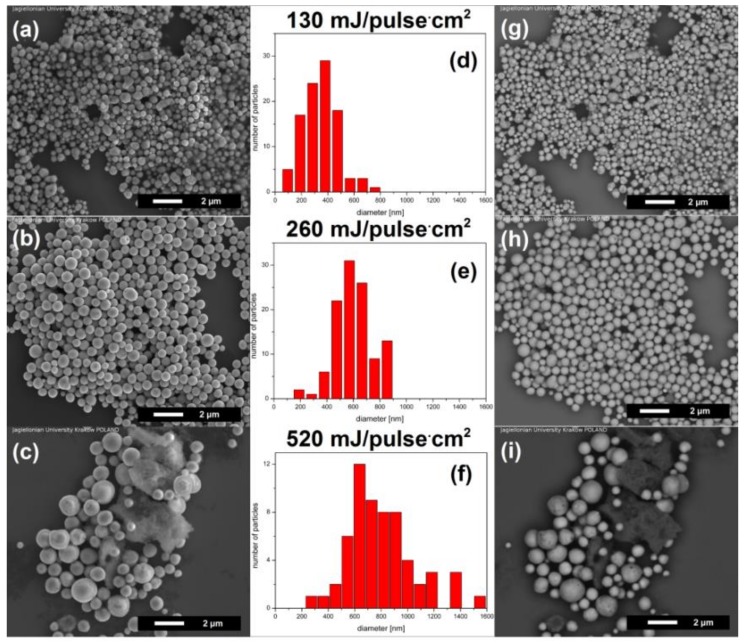
Submicron spherical particles obtained with pulsed laser irradiation of NiO nanoparticles dispersed in ethyl acetate with various laser fluence (laser wavelength of 532 nm, irradiation time—60 min): (**a**–**c**) SEM (scanning electron microscope) SE (secondary electrons) images (topography contrast); (**d**–**f**) particle size distribution histograms; (**g**–**i**) BSE (back scattered electrons) images (atomic number contrast).

Figure 1 shows that for the used laser fluencies and irradiation time of 60 min, the shape of newly formed submicron particles was always spherical while their diameter depended on the amount of deposited energy. For 60 min of irradiation we observed the particles with average diameter of 350 ± 70 nm, 580 ± 85 nm, 800 ± 130 nm for the fluence of 130 mJ/pulse·cm^2^, 260 mJ/pulse·cm^2^ and 520 mJ/pulse·cm^2^, respectively.

The back-scattered electron (BSE), visible at the right side of Figure 1, show atomic number contrast. Figure 1g shows areas with different contrast (bright and dark) which confirms that irradiation with low laser fluence (130 mJ/pulse·cm^2^) leads to creation of composite particles, while Figure 1h, for irradiation at 260 mJ/pulse·cm^2^, shows only bright colour, corresponding to the same composition in all directions of the particles. In Figure 1i the bright and dark colours indicate again existence of the composite spherical particles. However, in this case the spherical particles are accompanied by a very dark sponge with an irregular structure.

Additionally, we have investigated the structure of irradiated nanocomposites using an XRD method (Figure S2, Supporting Information). The raw nanoparticle diffractogram exhibits the diffraction peaks at 37.2° and 43.3° which correspond to (111) and (200) crystal planes of cubic NiO (JCPDS No. 71-1179) (Fm-3m) with a refined lattice constant of 4.187 ± 0.005 Å, as observed in bulk oxide. The big width of the diffraction peaks is related to the small diameter of raw particles. After irradiation at 130 mJ/pulse·cm^2^ (Figure S2b) the additional reflections at 44.5° and 51.9° appear in the diffractogram. They are attributed to (111) and (200) crystal planes of pure Ni (JCPDS No. 65-2865) (Fm-3m) with lattice constant a = 3.527 ± 0,004 Å. The positions of peaks from NiO are preserved and no changes in lattice constant are observed. The weight ratio of NiO/Ni, calculated from XRD data is 40:60. Further increase of the laser fluence to 260 mJ/pulse·cm^2^ led to an almost entire reduction of NiO manifested by the disappearance of corresponding peaks while the peaks of Ni were a little shifted to lower 2θ angles at positions of 44.06° and 51.22° (Figure S2c). This can suggest that into the cubic structure of Ni, the other element was incorporated. The careful analysis of the peak profiles indicated the presence of face-centered cubic(fcc)-NiC_x_ structure (space group Fm-3m) with lattice parameter a = 3.586 ± 0.005 Å slightly larger than that of fcc-Ni metal (3.524Å) [39], but noticeably smaller than that of fcc-NiC (4.077 A) [40]. The carbon content (x) was estimated from the lattice constant of NiC_x_ using the following relationship: a = 0.35447 + 0.00063 (at.%C) nm [41], which gave the value x = 0.06. The XRD patterns for particles prepared with larger laser fluences shows only the very broad reflections of NiC_x_ phase.

It is therefore seen that pulsed laser irradiation of NiO nanoparticles dispersed in ethyl acetate leads to the formation of particles which change the structure from Ni oxide to a mixed phase of NiO/Ni for 130 mJ/pulse·cm^2^ fluence, and next transform to NiC_x_ for larger laser fluences.

**Figure 2:**
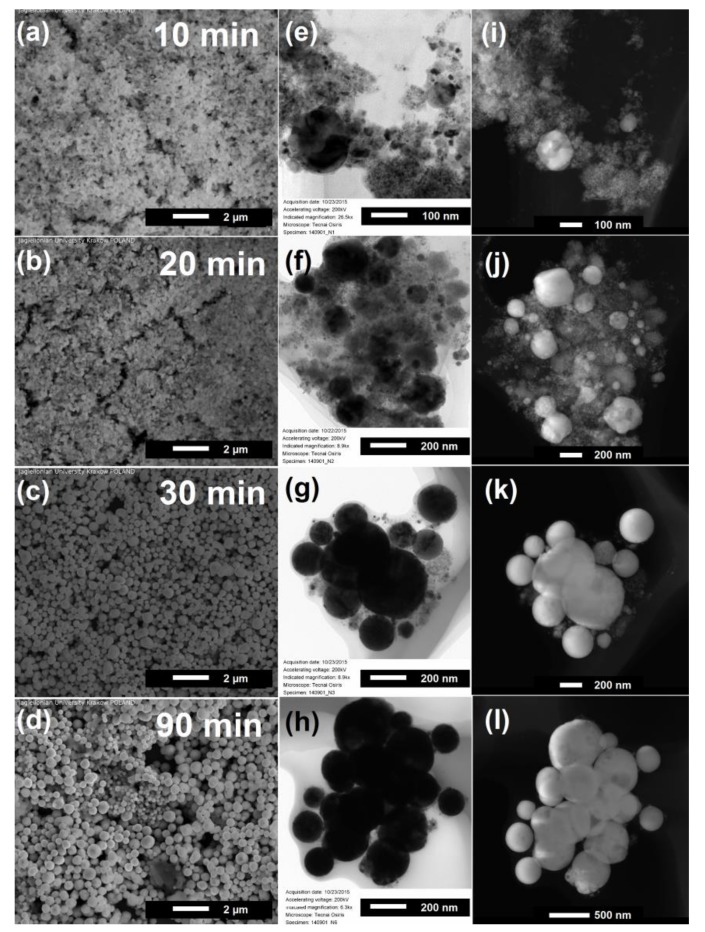
Electron microscopy images of particles prepared by pulsed laser irradiation (532 nm, 130 mJ/pulse·cm^2^) of NiO nanoparticles irradiated (**a**) 10 min; (**b**) 20 min; (**c**) 30 min; (**d**) 90 min: SEM SE (**a**–**d**); TEM (transmission electron microscope) (**e**–**h**); and STEM-HAADF (High angle annular dark field scanning transmission electron microscopy ) (**i**–**l**).

To get a deeper insight into transformations of nanoparticle composition during irradiation we performed their radiations with wavelength 532 nm and laser fluence of 130 mJ/pulse·cm^2^ as a function of irradiation time. Figure 2 illustrates the morphological changes of the particles for irradiation time in the range from 10 to 90 min. For the 10 min of irradiation we did not observe any changes to average particle size. The extension of irradiation time led to the appearance of sub-micrometer spherical particles and gradual increase of particles diameter. These observations clearly show a presence of time-dependent effects which influence the particle size, and indicate existence of heating–melting–solidification processes during pulsed laser irradiation [42,43].

TEM images provided more detailed structural information about the sub-micrometer spheres (Figure 2e–h). After 10 min of irradiation the particles have a diameter from a few nanometers to tens of nanometers. The increase of irradiation time to 30 min, resulted in the decrease of the number of small particles and the growth of particles with an average diameter of a few hundred nanometers. After 90 min of irradiation we obtained the homogeneous ensemble of particles with the average size of 360 ± 50 nm. STEM-HAADF results (Figure 2i–l) show the changes of particle composition with time. One can see that the amount of dark contrast places corresponding to NiO diminished with irradiation time in favor of the bight contrast places related to pure nickel. Simultaneously, this effect was accompanied by the increase of Ni particle diameter up to the value of a few hundred nanometers.

The similar dependence which showed gradual phase evolution of laser irradiation products was observed with XRD and can be found in Figure S3 of Supplementary Materials. The Ni phase appeared already after 20 min of irradiation and is present for the whole studied time range. However, with increase of irradiation time, the relative intensity of Ni peaks gradually increased, resulting in the 25% of NiO and 75% of Ni composition after 90 min of irradiation. 

**Figure 3:**
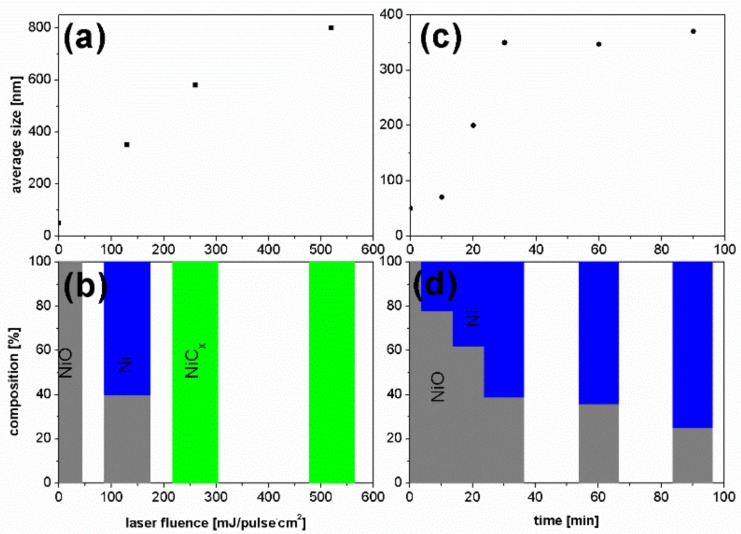
Dependence of particle (**a**) size and (**b**) phase changes on the laser light fluence (1 h of irradiation). Dependence of particle (**c**) size and (**d**) phase changes on irradiation time (130 mJ/pulse·cm^2^ fluence).

As can be seen from Figure 3a,c the size of particles increases gradually both with increase of laser fluence and time of irradiation. The particles sizes increased with fluence in the whole studied fluence range, while the time dependence was close to linear in the 10–30 min time range, and next saturates with the diameter of approximately 350 nm. The phase composition of particles known from XRD data (Figure 3b,d) follows a similar trend. For low laser fluence (130 mJ/pulse·cm^2^) a partial reduction of nickel oxide to metallic nickel occurs. Further increase of laser fluence leads to an entire reduction of NiO and formation of nickel carbide. The reduction of NiO to Ni during irradiation with constant fluence of 130 mJ/pulse·cm^2^ develops systematically during irradiation in the time range 10–30 min, but for longer irradiation time this process decelerates. These results demonstrate that after laser irradiation of NiO particles, choosing the proper combination of laser fluence and irradiation time, we obtain the composite particles being a mixture of NiO and Ni.

**Figure 4:**
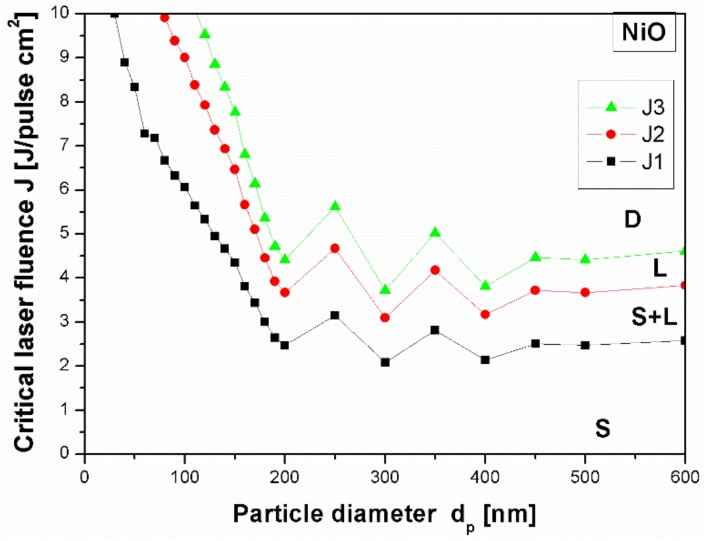
Theoretically calculated dependence of critical laser fluence on NiO particle diameter for different processes taking place during irradiation: the beginning of particle melting (J1), entire melting (J2), the decomposition (J3) (laser wavelength 532 nm). The capital letters S, S+L, L, D label regions of solid phase, mixture of solid and liquid phase, liquid phase and phase after decomposition, respectively.

From calculations shown in Figure 4 we see that the fluence of 130 mJ/pulse·cm^2^ is not big enough to start the process of nanoparticle melting. However, since the raw nanoparticles are small, they strongly agglomerate, adhering one to the other, which facilitates the energy transfer between them [28]. Without this aggregation process the formation of larger particles is not possible for irradiation conditions used in our experiment. The agglomerated particles need smaller energy for melting and decomposition leading to the formation of spherical composites. We demonstrated the creation of spherical composite particles at small energy fluence which means that in the experimental conditions, the aggregation described above takes place. 

The difference between the calculated and experimental energy value can be explained not only by agglomeration of the particles, but also by the pyrolysis process of ethyl acetate which most likely introduces into solution ions or molecules that absorb light and transmit the absorbed energy to NiO. It is known that the carbon can enhance the absorption of low optical-absorption materials like Al_2_O_3_, MgO and ZrO_2_ due to high optical absorption of laser light [30]. We have already observed such an effect for nickel oxide irradiated with a single laser pulse in the presence of gold which favoured energy transfer from Au to NiO causing it to melt and form spherical particles [35]. During pulsed irradiation by repeating the processes of nanoparticle agglomeration, laser energy absorption and melting–solidification of particles, the particles grew in size [28]. Therefore, we think that carbon created by the pyrolysis process and present in synthesized composites play similar roles. The investigation of the role of solvent in the process of particle growth requires additional experimental and theoretical works.

**Figure 5:**
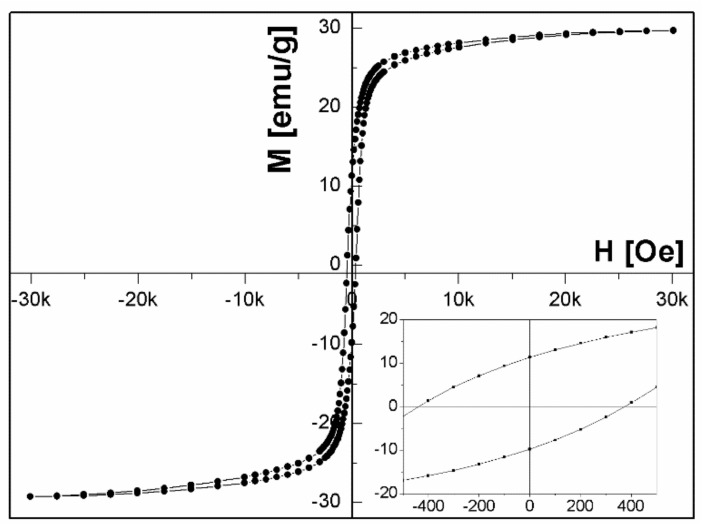
Hysteresis loop of NiO/Ni particles fabricated at 130 mJ/pulse·cm^2^ for 20 min. Inset: The magnification around origin of hysteresis loop.

We studied also the magnetic properties of Ni/NiO composite particles. The bulk NiO is an antiferromagnet while Ni is a ferromagnet. The exchange bias, which is an interfacial effect of the exchange coupling between antiferromagnetic (AFM) and ferromagnetic (FM) material, induces a unidirectional anisotropy of the ferromagnet and should be expected in NiO/Ni systems. We measured the hysteresis loops at 5 K after cooling the sample in magnetic field of 30 kOe for the particles irradiated with laser fluence of 130 mJ/pulse·cm^2^ and different irradiation time. Figure 5 illustrates the typical loop for the irradiation time of 20 min.

**Figure 6:**
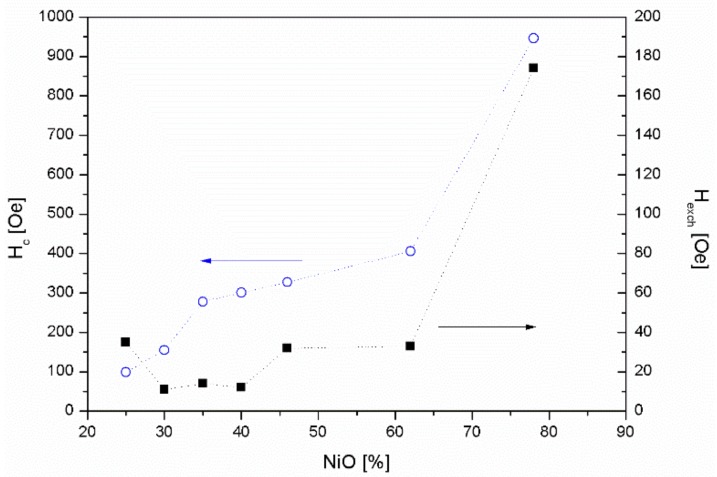
Coercivity H_C_ and exchange bias H_EB_ of NiO/Ni composite particles as a function of relative fraction of NiO.

The exchange bias field is defined as a loop shift H_EB_ = |(H^+^ + H^−^)/2|, where H^+^ and H^−^ are positive and negative fields for which the magnetization is equal to zero, while the coercive field is given by H_C_ = (H^+^− H^−^)/2. Figure 6 shows the variation of coercive H_C_ and exchange bias H_EB_ fields as a function of NiO content in NiO/Ni particles measured at 5 K after cooling in field of 30 kOe. As can be seen, the values of both H_EB_ and H_C_ change in a similar way. They increase with the increasing NiO fraction reaching a maximum of 950 Oe for coercive field and exchange bias field of 175 Oe for 80% of NiO fraction. H_EB_ and H_C_ growth with the increase of NiO fraction is similar to that reported by others [25,27]. It is worth pointing out that the H_EB_ and H_C_ obtained for samples with 80% of NiO fraction are higher than reported by Yao et al. [27], for samples with the same concentration of NiO, prepared by chemical method. Similarly, for particles containing 74% of NiO presented by Querejeta-Fernández et al. [25], the H_EB_ and H_C_ are lower than for particles with the same content of NiO obtained by laser irradiation. This could mean that pulsed laser irradiation maximizes the extent of NiO/Ni interfacing.

For the NiO weight fraction below 80%, the effect of interactionsat ferromagnetic and antiferromagnetic interfaces is very weak, and hence we observed smaller values of exchange bias field. For NiO weight fraction of 80%, the effect of interfaces becomes dominantand results in the enhancement of exchange interactions. 

As also can be seen from Figure 6 the coercive field H_C_ increases the value for the same content of NiO which could be related to the instability of antiferromagnetism and domain-wall pinning [44,45].

In general, exchange bias fieldis sensitive to numerous factors, such as interfacial roughness, dimensions of both the antiferromagnet and ferromagnet, AFM and FM domain size, and AFM anisotropy energy [46,47,48,49,50]. All these parameters can affect the number of pinned/unpinned uncompensated spins at the AFM/FM interface.

**Figure 7:**
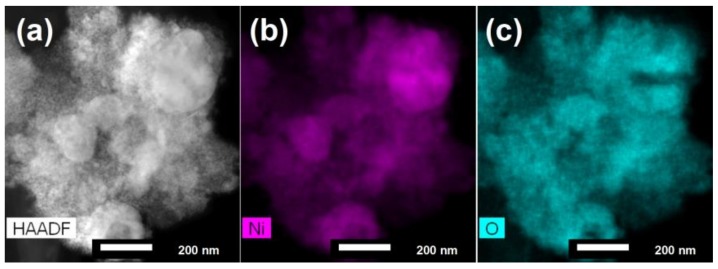
(**a**) HAADF image, and (**b**,**c**) STEM-EDX mapping results for Ni and O elements of particles obtained with pulsed laser irradiation with irradiation time 10 min.

The distribution of elements in the particles obtained with laser irradiation and with irradiation time of 10 min was investigated by elemental mapping using EDS coupled with STEM. In the HAADF-STEM (Z contrast) image (Figure 7a), bright areas correspond to the Ni particles whereas faint grey areas indicate the presence of the less heavy scatterers (NiO), as additionally confirmed by EDXS mapping (Figure 7b,c). These results show that the majority of samples consist of small NiO particles with only a few places of large Ni areas. These areas most likely create the Ni/NiO interfaces responsible for observed exchange bias field.

## 4. Conclusions

In conclusion, the pulsed laser irradiation technique was demonstrated to be a simple method for preparation of submicron NiO/Ni heterostructure particles. We showed that by varying the laser parameters (laser fluence and/or irradiation time), we can control the phase ratio of Ni and NiO. We have characterized the structure, morphology and magnetic properties of submicron spherical particles and observed the creation of heterojunctions at the interfaces composed of fcc NiO and fcc Ni, a mixture which induced the presence of exchange bias effect. The presence of approximately 20% of Ni weight fraction resulted in the increase of coercive and exchange bias fields with the value at 5 K being 950 Oe and 175 Oe, respectively. We showed that by varying the phase ratio of Ni and NiO, we can control the coercivity and exchange bias effect.

We believe that laser irradiation of NiO nanoparticle colloids could open new possibilities for synthesis of heterostructure particles with controllable size, composition and magnetic properties, and can be important for fabricating nanocomposite particles, due to its simplicity, controllability, and contamination-free nature (pulsed laser irradiation method does not require chemicals or stabilizers for synthesis nor for particle stabilization).

## Supplementary Materials

The following are available online at http://www.mdpi.com/2079-4991/8/10/790/s1, Figure S1: SEM image and SEM EDS analysis result of raw NiO particles, Figure S2: X-ray diffraction patterns of raw NiO nanoparticles and products obtained with laser irradiation of NiO nanoparticles at various laser fluences 130 mJ/pulse·cm^2^, 260 mJ/pulse·cm^2^, 390mJ/pulse·cm^2^, 520 mJ/pulse·cm^2^ (532 nm, 60 min), Figure S3: X-ray diffraction patterns of raw nanoparticles and products obtained with laser irradiation of raw NiO nanoparticles at various irradiation time 10 min, 20 min, 30 min, 40 min, 50 min, 90 min, Ni, NiO (532 nm, 130 mJ/pulse·cm^2^), Figure S4: SEM image, BSE image of submicron spheres particles obtained with pulsed laser irradiation of NiO nanoparticles dispersed in ethyl acetate with various irradiation time: 10 min, 20 min, 30 min (532 nm, second harmonic, 390 mJ/pulse·cm^2^), Figure S5: Image of submicron spheres particles obtained with pulsed laser irradiation of NiO nanoparticles dispersed in ethyl acetate with various irradiation time: 10 min, 20 min, 30 min (532 nm, second harmonic, 390 mJ/pulse·cm^2^), Table S1: Coefficients of refraction n and extinction coefficients k for NiO, Table S2: Thermodynamic and chemical processes with NiO as initial material.
