# Supplementary material for: Tailoring of Magnetic Properties of NiO/Ni Composite Particles Fabricated by Pulsed Laser Irradiation
Source: Nanomaterials (Basel). 2018 Oct 5;8(10):790. doi: 10.3390/nano8100790 (PMC6215229; doi:10.3390/nano8100790)
Supplement: Supplementary file 1 [file nanomaterials-08-00790-s001.pdf]

Supporting information

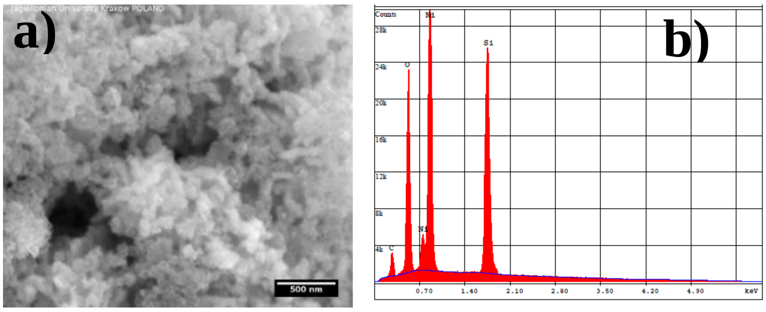

Figure S1. (a) SEM image and (b) SEM EDS analysis result of raw NiO particles.

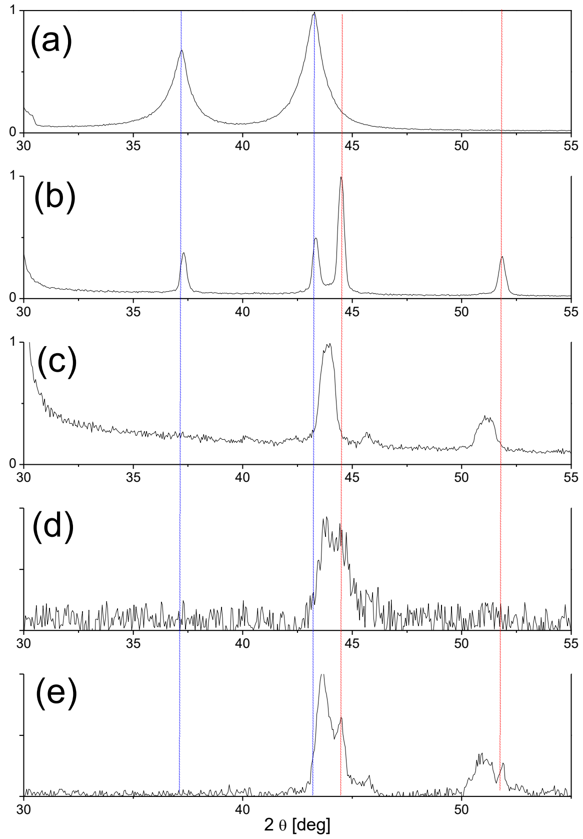

Figure S2. X-ray diffraction patterns of (a) raw NiO nanoparticles and products obtained by laser irradiation of NiO nanoparticles at various laser fluences (b) 130 mJ/pulse·cm^2 (c) 260 mJ/pulse·cm^2, (d) 390mJ/pulse·cm^2, (e) 520 mJ/pulse·cm^2 (532 nm, 60 minutes). Standard XRD peaks for NiO (red line) and Ni (blue line) are plotted for reference.

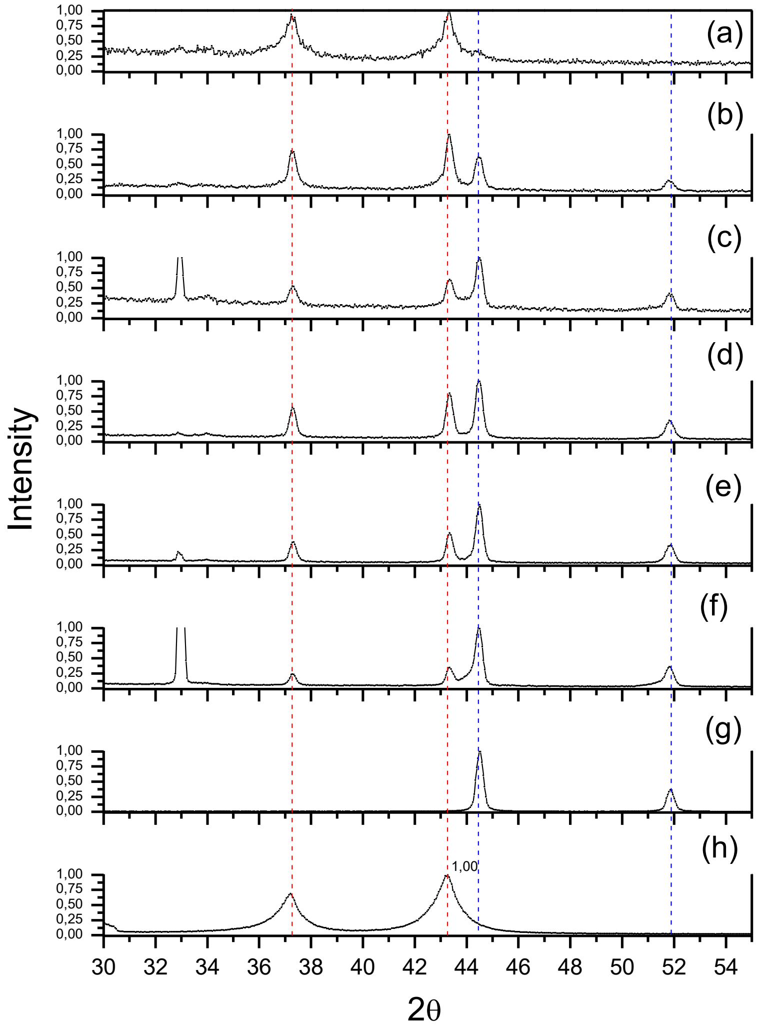

Figure S3. X-ray diffraction patterns of raw nanoparticles and products obtained by laser irradiation of raw NiO nanoparticles at various irradiation time (a) 10 min, (b) 20 min, (c) 30 min, (d) 40 min, (e) 50 min, (f) 90 min, (g) Ni, (h) NiO(532 nm, 130 mJ/pulse·cm^2). Standard XRD peaks for NiO (red line) and Ni (blue line) are plotted for reference.

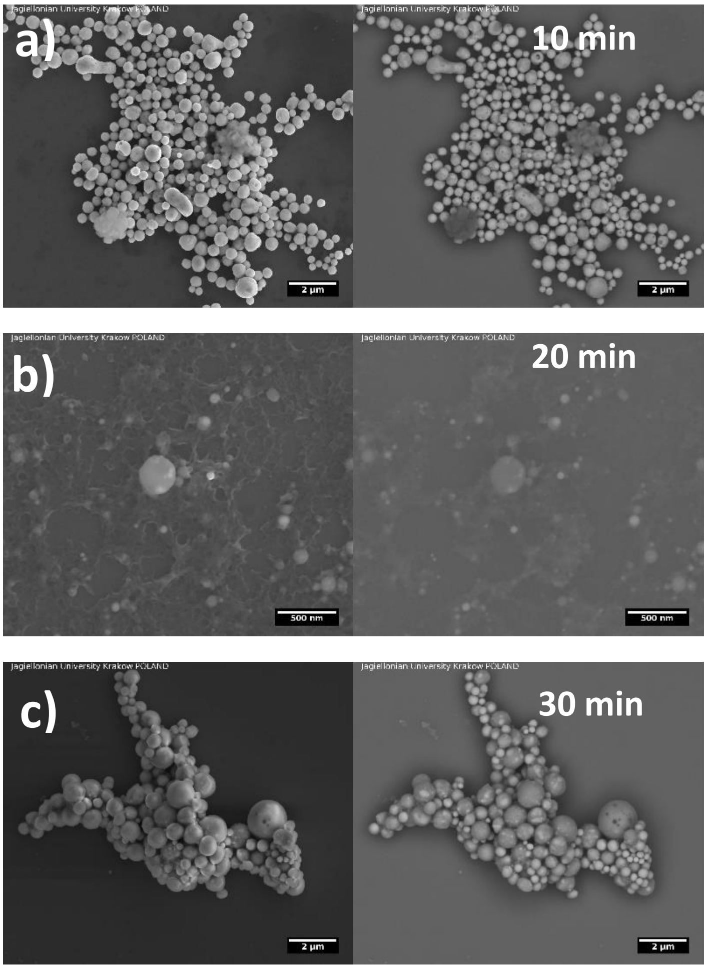

Figure S4. (left) SEM image, (right) BSE image of submicron spheres particles obtained by pulsed laser irradiation of NiO nanoparticles dispersed in ethyl acetate with various irradiation time: (a) 10 min, (b) 20 min, (c) 30 min (532 nm, second harmonic, 390 mJ/pulse·cm^2).

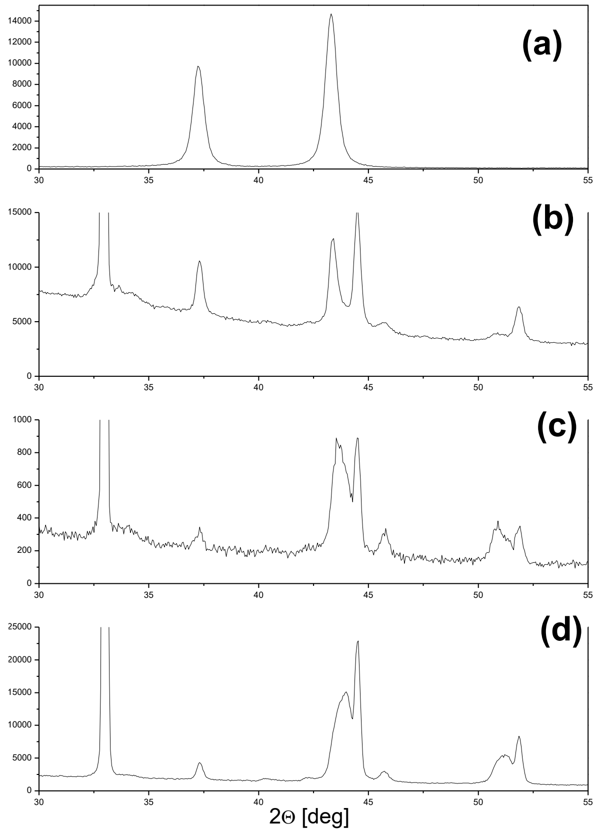

Figure S5. Image of submicron spheres particles obtained by pulsed laser irradiation of (a)NiO nanoparticles dispersed in ethyl acetate with various irradiation time: (b) 10 min, (c) 20 min, (d) 30 min (532 nm, second harmonic, 390 mJ/pulse·cm^2).

TableS1. Coefficients of refraction n and extinction coefficients k for NiO.[1,2]

λ=532 nm		
	n	***k***
**NiO**	2.317	0.009

Table S2. Thermodynamic and chemical processes with NiO as initial materials.[2,3]

Reaction	Process	T [K]	ΔH(T) [kJ/mol]
NiO melting	NiO(c)=NiO(l)	2228	54.4
NiO decomposition	NiO(l)=Ni(l)+1/2O_2	2848	193.6
Ni evaporation	Ni(l)=Ni(g)	3159	378

Literature:

1. Henley, S. J., Carey, J. D., Silva, S. R. P. Pulsed-laser-induced nanoscale island formation in thin metal-on-oxide films, *Phys. Rev. B* **2005**, *72*, 195408.
2. Swiatkowska-Warkocka, Z.; Pyatenko, A.; Koga, K.; Kawaguchi, K.; Wang, H.; Koshizaki, N. Synthesis and Control of Various Morphologies/Phases of Au-based Nanocomposite Particles by Pulsed Laser Irradiation in Liquid Media, *J. Phys. Chem. C*, **2017***121*, 8177-8187.
3. JANAF thermochemical tables 1974 supplement.
